# Developing a comparative photon‐proton planning service in Victoria: the experience at Peter MacCallum Cancer Centre

**DOI:** 10.1002/jmrs.754

**Published:** 2024-02-02

**Authors:** Roshini Gunewardena, Lisa Hall, Michelle Li, Gabrielle Drum, Dianna Le, Emily Nigro, Bridget Houlder, Claire Phillips, Greg Wheeler, Kirsty Wiltshire, Tomas Kron, Adam Yeo

**Affiliations:** ^1^ Peter MacCallum Cancer Centre Melbourne Victoria Australia; ^2^ Sir Peter MacCallum Department of Oncology University of Melbourne Melbourne Victoria Australia; ^3^ School of Applied Science RMIT University Melbourne Victoria Australia

## Abstract

Proton‐beam therapy (PBT) is a cutting‐edge radiation therapy modality that is currently not available in Australia. Comparative photon‐proton (CPP) planning is required for the medical treatment overseas programme (MTOP) and will be required for access to PBT in Australia in the future. Comparative planning brings professional development benefits to all members of the radiation therapy team. This service was also created to support future proposals for a PBT facility in Victoria. We report our experience developing an in‐house CPP service at Peter MacCallum Cancer Centre. A set of resources to support CPP planning was established. Training of relevant staff was undertaken after which an in‐house training programme was developed. A standard protocol for PBT planning parameters was established. All CPP plans were reviewed. Future goals for the CPP planning programme were described. In total, 62 cases were comparatively planned over 54 months. Of these, 60% were paediatric cases, 14% were adolescents and young adults (15–25 years) and 26% were adults. The vast majority (over 75%) of patients comparatively planned required irradiation to the central nervous system including brain and cranio‐spinal irradiation. A variety of proton plans were reviewed by international PBT experts to confirm their deliverability. Our team at Peter MacCallum Cancer Centre has gained significant experience in CPP planning and will continue to develop this further. Local expertise will help support decentralisation of patient selection for proton treatments in the near future and the PBT business case in Victoria.

## Graphical abstract

Comparative planning brings professional development benefits to all members of the radiation therapy team. This service was also created to support future proposals for a Proton‐beam therapy facility in Victoria. We report our experience developing an in‐house comparative photon‐proton planning service at Peter Mac.

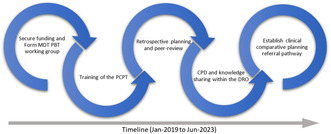

## Background

Proton‐beam therapy (PBT) is a cutting‐edge radiation therapy modality available in only 95 facilities globally as of today.^1^ The conformity of radiation dose to tumours achievable with proton‐beams can result in less radiation to healthy tissues, compared to standard photon radiation therapy (XRT).^2^ As such, PBT has the potential to improve long‐term health outcomes for children, adolescents and young adults (AYA) with cancer, and adults with difficult‐to‐treat cancers.^3^

Across the globe, the number of PBT centres has grown exponentially,^4^ reflecting recent, rapid technological improvement in PBT and the growing evidence base on the health, social and economic benefits of PBT. It is anticipated that there will be an increase in the number of patients with cancer who may be considered for PBT. However, PBT is not currently available in Australia due to the scale and cost of beam delivery systems. Construction of the Australian Bragg Centre for Proton Therapy and Research (ABCPTR) is underway in Adelaide and expected to open in mid‐2025.^5^ Currently, Australian patients wanting to access Proton Therapy must self‐fund or successfully apply for treatment under the medical treatment overseas programme (MTOP). The MTOP application process requires a photon‐proton comparison plan that supports the clinical decision to undergo PBT overseas rather than photon treatment in Australia. It has been proposed that there will be a similar process once PBT becomes available in Australia. As a result, the team at Peter MacCallum Cancer Centre felt there was a need to develop education and expertise in this modality to enable better discussion with patients regarding the option of PBT and workforce development.

Peter Mac is Australia's only public hospital solely dedicated to cancer and the sole provider of paediatric radiation oncology services in the state of Victoria and Tasmania, treating approximately 150–200 paediatric and AYA patients each year. In Victoria, 37% of new cancer patients in Victoria receive radiation therapy (RT) whilst up to 50% of cancer patients would ideally receive a form of RT at some time.^6^ PBT is considered preferential when a meaningful reduction in dose to surrounding healthy tissue can be achieved compared with conventional photon treatment^7^; such a difference is estimated to benefit 5–15% of all radiation therapy patients.^8^

The Peter Mac journey building the comparative photon‐proton (CPP) planning service was conceived in 2017. Since then, it has become evident that there is a need to form a core working group within the Division of Radiation Oncology (DRO) to build clinical knowledge and practical experience, such as; PBT equipment/beam characteristics, PBT planning, and its evaluation in comparison with photon plans. In this context this report details the development of an in‐house CPP planning service. We share the current status of our clinical experience to help other providers create their own.

## Development process of comparative planning service

**Figure 1:**
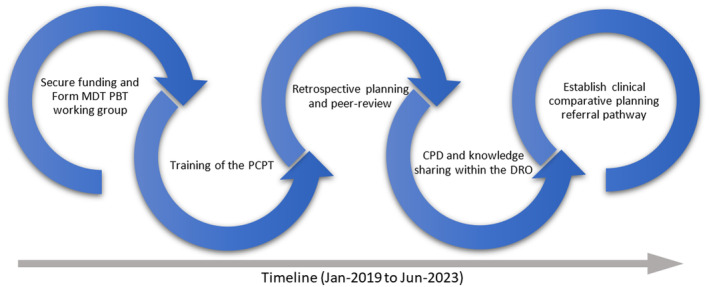
A schematic of development of in‐house photon‐proton comparative planning service. A list of abbreviations: MDT – Multidisciplinary team, PBT – Proton‐beam therapy, PCPT – the Photon‐proton Comparative Planning Team, CPD – Continuing Professional Development, and DRO – the Division of Radiation Oncology.

This section describes how an in‐house CPP planning service was established over a period of 4.5 years (January 2019 to June 2023). In keeping with our current photon treatment planning service, the Photon‐proton Comparative Planning Team (PCPT) is a radiation therapist (RT) led service. The team also includes a lead radiation oncology medical physicist (ROMP) and radiation oncologists (RO). The development process is summarised in Figure 1 as a schematic, illustrating that the five steps are sequential in general, but each step is overlapped with its subsequent step in timeline. The details of each step in the timeline are described in the following discussion.

### Secure funding and formation of multidisciplinary team (MDT) working group

Philanthropic and state government funding was obtained by our institution for PBT training as well as research and development (R&D). Funding from the Peter Mac Foundation in DRO enabled specific PBT training for a medical physicist allowing two site‐visits at National Cancer Centre in South Korea over the last 4 years. Funding from the Department of Health and Human Services Victoria enabled secure planning time (0.1 Full‐time equivalent) as well as CPD (Continuing Professional Development) opportunities shared among three radiation therapists. A radiation oncologist was awarded the RANZCR Thomas Baker Fellowship and spent a year at The Christie PBT centre. Expressions of interest (EOI) were sought from RT craft group who had demonstrated an interest in PBT and had experience in paediatric RT planning.

### Training of the PCPT

The aforementioned funding and development activities of the PCPT have enabled the creation of training resources and the opportunity to foster relationships with experienced PBT clinics overseas that are equipped with relevant treatment planning system (TPS) and PBT delivery systems for our comparative planning practice implementation. The overseas opportunities of members of the PCPT have enabled us to learn first‐hand from the experiences of other centres through their development phases in PBT. Further to this, the experience of our ROMP led to validation of the beam model utilised in our PBT comparative plans and consolidation of our plan evaluation processes with various dose calculation algorithms. In‐service training as well as vendor training was provided to the PCPT. A monthly PBT journal club was established as a forum for a wider group of interested staff members to share knowledge and discuss latest research findings in the area.

### Retrospective planning and peer‐review

The development of the planning skillsets within our RT workforce has provided the foundation on which we have built our in‐house CPP planning service. From the appointment of the RT proton group in early 2019, patient plans were retrospectively re‐planned using our newly validated TPS proton beam model with an initial focus on paediatric and neuro‐oncology cases as per our primary interest from the clinical team for future PBT indications and its potential referrals. This progressively expanded to encompass a broad range of anatomical sites. These plans were externally reviewed during PBT workshops conducted by the Trans‐Tasman Radiation Oncology Group (TROG), by experienced international PBT facilities using web‐based platforms, as well as face‐to‐face sessions with visiting clinicians and colleagues from The Christie, University College London Hospital (UCLH) and Massachusetts General Hospital (MGH). This review process was undertaken for a range of body sites including cranio‐spinal, breast and neuro‐oncology cases and provided invaluable feedback on proton plan quality produced by the PCPT. For selected cases, proton comparative plans were concurrently completed by the more experienced ABCPTR team in South Australia for the purpose of application to MTOP. In these situations, a side‐by‐side review was completed of our in‐house proton plan with that completed by ABCPTR to help inform our planning strategy and create opportunities for further development and learning. A comparative planning template was adopted from ABCPTR and we added a few more parameters such as the conformity index (CI100 and CI50) as well as for range uncertainty parameters for documentation purposes. It is important to note that there was a slow build‐up (of skills and development process) from January 2019 to January 2021, which led to an active CPP planning phase thereafter.

### CPD and knowledge sharing within the DRO

Concurrent to this work, CPD activities have been undertaken as a priority to consolidate the experience of the group. Examples include: The Christie Proton Therapy e‐school, MD Anderson Proton Head and Neck (H&N) Webinar and conference attendance such as Particle Therapy Co‐Operative Group (PTCOG), and annual National Particle Therapy Symposium (NPTS). The resources gained from these activities was consolidated and shared among the PCPT and the wider department through staff meetings, proton journal club, and on online resource platforms. The initial group of three RT members also grew to expand the service capacity and allow for greater flexibility in balancing clinical service delivery in our busy six linear accelerator department. In addition to relevant information sharing among peers within DRO, the PCPT has also been actively sharing PBT knowledge and experience to those outside of Peter Mac, through forums such as;

- PTCOG Conference 2021 and 2022 with a presentation on machine‐learning based planning and machine utilisation simulation
- NPTS, Melbourne 2020, which included the TROG Particle Therapy Special Interest Group (PTSIG) planning workshop
- Australian Society of Medical Imaging and Radiation Therapy (ASMIRT) 2021: Building Local Proton Beam Therapy Planning Knowledge in an Advanced Paediatric Radiation Therapy Centre

### Establish clinical comparative planning referral pathway

In developing a clinical pathway for CPP planning referrals since January 2021, it was intended that our workload management should mirror that which is currently in place for radiotherapy electronic treatment requests for photon, superficial and Gamma Knife radiotherapy treatments. Utilising the MOSAIQ® record and verify system, work has been undertaken to develop an electronic request for CPP planning services. This form generates quality check‐list items that can be used to triage and prioritise the CPP planning workload. Additional data points that are pertinent to the PBT planning process have been included, such as margins used for clinical target volumes and planned target volumes. The pathway has been designed so that the photon planning component is retained by the relevant tumour stream planning group, as per our standard practice. Upon receipt of a proton comparison request, the PCPT meets with the prescribing clinician to discuss planning goals and document relevant considerations. The PCPT discusses at this pre‐planning meeting potential beam arrangement and technical aspects of the plan, before one RT is tasked with completing the plan. Once peer‐reviewed within the PCPT group and compared to the photon plan, the plan is sent to the prescribing RO for review.

## A summary of comparative photon‐proton planning

### Beam models and calculation settings

All proton plans were created using the Varian pro beam model data on the Eclipse TPS (Eclipse™ v16.1, Varian Medical Systems, Palo Alto, CA, USA) and compared to a photon volumetric modulated arc therapy (VMAT) plan as per our standard clinical practice (Table 1). Proton planning utilises Intensity Modulation Proton Therapy (IMPT) using spot‐scanning technique and inversed plan optimisation. The beam models employed a constant dose scaling factor by 10% to apply a fixed RBE of 1.1, which is adopted as a pragmatic clinical standard around the globe.^9^ Calculation models and detailed settings used in the CPP planning are summarised below. Use of GPU and heterogeneity correction options are enabled for both photon and proton final dose calculations as well as optimisation processes.

**Table 1:** A summary of Calculation models and detailed settings used in the photon‐proton comparative planning.

Eclipse TPS (v16.1)	Photon model settings	Proton models settings
Machine model	Varian TrueBeam	Varian ProBeam
Final dose calculation	AcurosXB_1610	AcurosPT_1610
Dose reporting mode	Dose‐to‐medium	Dose‐to‐medium
Optimiser	Photon Optimiser (PO)_1610	Nonlinear universal proton optimizer (NUPO)_1610
Dose optimisation algorithm	Multiresolution dose calculation (MRDC)_1610	Proton convolution superposition (PCS)_1610
Beam‐line modifier	N/A	Proton convolution superposition (PCS)_1610
Energy layer/Spot spacing	N/A	Spot size in Air FWHM^1^
Dose Grid (cm)^2^	0.25 or 0.125	0.25
Margins or Range uncertainty parameters (TPS specific)^3^	5–10 mm isotropic PTV margins	3.0–5.0% CT curve error 1‐2 mm additional proximal/distal margins 2‐3 mm setup errors

^1^ Energy‐layer dependent in‐air FWHM size at isocentre, typically ranging from 3 to 9 mm for 70 MeV‐250 MeV energy range [Ref 13].

^2^ Dose grid size is constant for both optimisation and final dose calculation processes. For photon planning, standard dose grid‐size is 0.25 cm whereas 0.125 cm is used for stereotactic treatment regimes.

^3^ Range uncertainty parameters are TPS specific. A typical CT calibration curve error is ±3.5% ranging from 3% (e.g. brain) to 5% (e.g. lung). A range of additional safety margins are also applied depending on treatment sites in terms of setup stability, beam pathlength and heterogeneity.

### Planning approach

For proton planning, standard field arrangements were used utilising up to a maximum of four beams. Beam specific targets were created and a universal range shifter of 5 cm applied when the CTV extended to the skin surface. Working in a similar way to build up material in photon planning to improve target coverage.

CTV‐based robust optimisation was used, and a set of range uncertainty parameters were applied (which is TPS specific) depending on treatment sites. Typically, ±3.5% CT calibration error (varying from 3% for brain to 5% for lung as examples), 2–3 mm setup error and 1‐2 mm of additional proximal and distal margins (for field‐specific target generation), which serve as additional site‐specific safety margins depending on treatment sites in terms of setup stability, beam pathlength and heterogeneity (refer to Table 1). Multi‐field optimisation was utilised for most cases with single‐field optimisation considered in others. Plan uncertainty parameters were calculated and robust plan evaluation was undertaken utilising the equivalent robustness parameters used during robust optimisation. In addition to the nominal plan, at least 12 plans under the different uncertainty scenarios were created to assess the worst‐case scenarios in relation to the geometry of targets and adjacent OARs. It is worth noting that this CTV‐based robust planning is in line with the guideline^12^ and the process of quality control emulates a standard practice of our photon planning including second RT check and physics plan check/QA. Indeed, it was somewhat more stringent as photon‐proton plan comparisons were reviewed by PCPT as a team.

**Figure 2:**
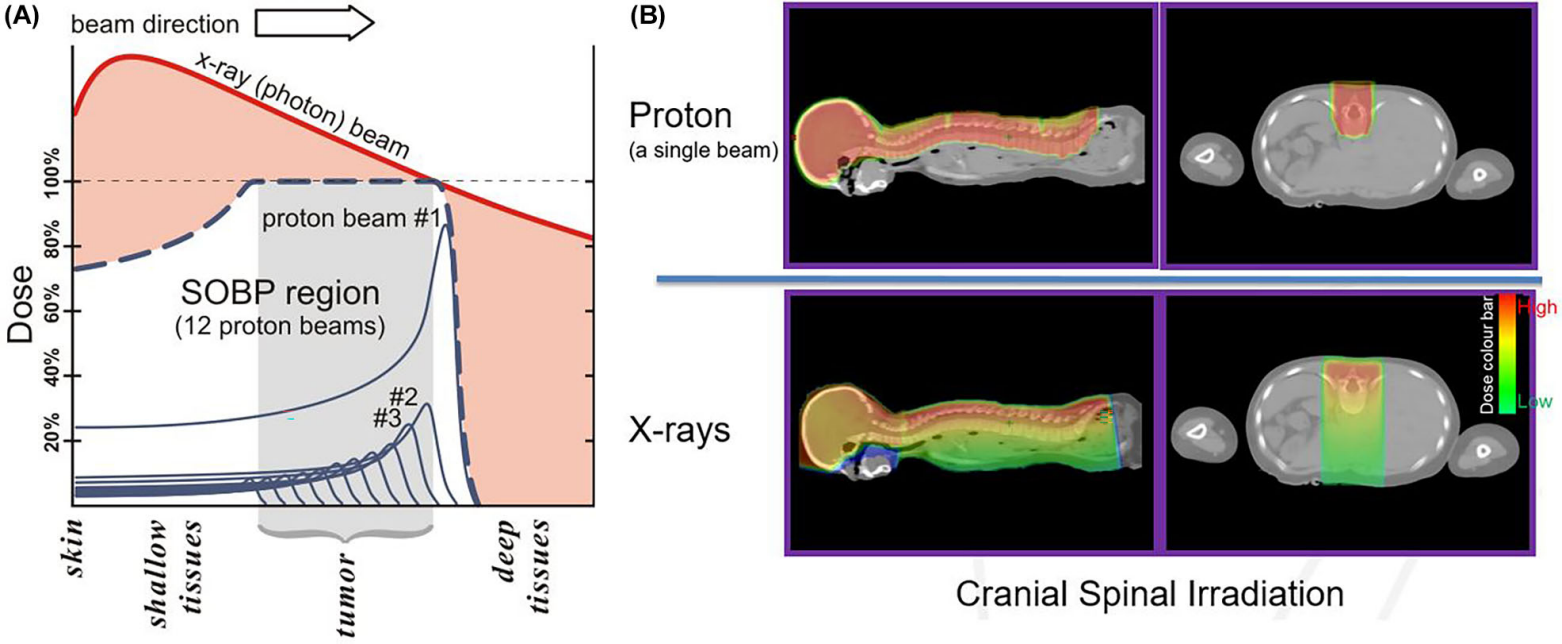
(A) A comparison x‐ray photon radiation therapy (XRT) and proton‐beam therapy (PBT) in terms of radiation dose deposition to spine and brain for paediatric cranio‐spinal irradiation (generated by Peter Mac PBT team); (B) a single beam comparison of proton‐photon depth dose curve including spread‐out Bragg‐peak (SOBP) for illustration purpose.

Figure 2A shows a comparison of percentage depth dose between a proton beam and a photon beam, illustrating the difference in dose fall‐off through beam direction. Proton beam uses multiple beam energies to deposit dose in different layers, starting with distal part of the tumour with higher beam weighting then move to proximal direction with lower beam weighting. This Figure shows 12 proton beams to form a spread‐out bream peak (SOBP) to cover the entire target. Figure 2B shows an example of single‐beam dosimetric comparison of the two plans in context of cranio‐spinal irradiation (CSI) for illustration purposes only. Field‐by‐field robust optimisation (RO) is employed to reduce the extent of dose‐gradient at field junctions.

### Planning cases

A total of 62 comparative planning cases were collated over 54 months for retrospective analysis. Table 2 shows the yearly breakdown with a consistent caseload, just over one case each month throughout the period. A two‐phase approach was taken for a smooth transition – Phase 1: 47% (*n* = 29) with a mix of training and clinical development cases (over 2019–2020) then Phase 2: 53% (*n* = 33) based on clinicians' request (2021 onward).

**Table 2:** Yearly breakdown of the number of cases and its average number of cases per month. Average number of cases each year is consistently around 1.15 with standard deviation of 0.15.

Yearly breakdown (# of months)	# of cases per year	Avg. cases/month
2019 (12 months)	12	1.00
2020 (12 months)	17	1.42
2021 (12 months)	12	1.00
2022 (12 months)	14	1.17
2023 (6 months)	7	1.17
Total	62	1.15 (SD = 0.15)

**Figure 3:**
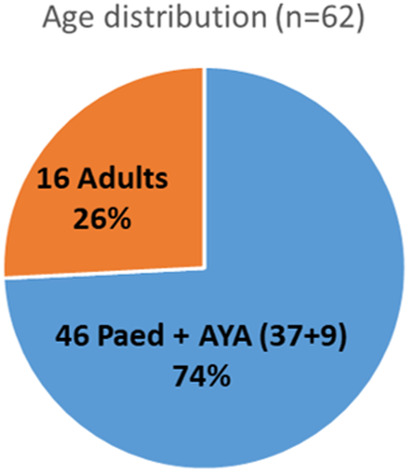
A summary of total number of cases with breakdown in terms of patient ages: (i) paediatric (<15 years); (ii) Adolescent/Young‐Adult (AYA, 15 years–25 years) and (iii) adults (>25 years).

Figure 3 shows the total case in terms of age distribution: 60% (*n* = 37), 14% (*n* = 9) and 26% (*n* = 16) for paediatric <15 years, AYA between 15 and 25 years and adults >25 years, respectively, illustrating the heavy focus on the paediatric cohort.

Table 3 shows a more detailed breakdown of distribution based on tumour location. The majority (77.4%, *n* = 48) were from the central nervous system (CNS) and comprises of focal brain (*n* = 38) and CSI (*n* = 10) treatments. Whereas for the adult cohort, CNS includes brain only (62.5%, 10 out of 16) and there was no CSI indication. The remaining cases (26%) is comprised of H&N (14.5%, *n* = 9), pelvis (4.8%, *n* = 3) and breast (3.2%, *n* = 2) sites. Of note, eight proton plans (13% of total cases) across different body sites such as brain, CSI, H&N and breast were reviewed by international PBT clinics to confirm its deliverability. DICOM data (including RTstructure, RTplan, RTdose) as well as plan summary report were de‐identified and transferred for the peer‐review process.

**Table 3:** Case breakdown by different age groups and body sites for treatment planning.

Patient cohorts by age	# of cases	% ratio	Body sites	# of cases
Paeds (Age < 15)	37	59.7%	CNS (Brain+CSI)	38 (28 + 10)
AYA (15 ≤ age ≤ 25)	9	14.5%	H&N	5
Total (Paeds + AYA)	(46)	(74.2%)	Pelvis	3
			CNS (Brain)	10
Adults (Age > 25)	16	25.8%	H&N	4
			Breast	2

A list of abbreviations: Paeds – Paediatric patient cohort, AYA – Adolescent and Young‐adult patients cohort, CNS – Central Nervous System, CSI – Cranio‐spinal Irradiation, and H&N – Head & Neck patients cohort.

## Discussion

### Challenges and limitations

Since the formation of the PCPT service at our institution, 62 cases have been comparatively planned. Over the first 2 years, focus was given to building the planning skillset and experience through retrospective planning of clinical cases of interest, initially focusing on paediatric cancers and adult CNS cancers.

Due to the global pandemic, training was limited to local and online, and reduced opportunities to gain practical experience at PBT facilities. As a result, online meetings with overseas PBT teams were helpful to identify plans that would be challenging to deliver. In hindsight, establishing a partnership with an experienced overseas PBT facility earlier in the service development would have been beneficial.

Another challenge currently facing the service is the limited resources to accommodate for increased interest in PBT among DRO clinicians. After the introduction of the monthly PBT journal club, clinician requests for CPP have increased. As a result, there has been a need to triage based on indication (clinical need, education or research) to help balance allocation of resources. With the upcoming availability of PBT in Australia, this increasing demand highlights the need for further funding across the MDT, to ensure ongoing training, including site visits to operational PBT facilities overseas and expansion of the PCPT.

### Future considerations

Based on the current treatment workload, our workforce prediction model estimates that PBT would currently be suitable for approximately 60% of paediatric and 50% of AYA cancer patients.^10^ This indicates about 50 cases for each cohort (in total 100 in 2025) in terms of Victoria‐wide demand, which is expected to show a steady increase with 5–8% yearly increments with potential expansion of approved indications for PBT in coming years.^11^, ^12^ Our experience in planning with PBT would benefit a smoother transition if a business case for a PBT facility in Victoria is successful in the near future.

Without a PBT facility in Australia, MTOP is the only funded pathway to refer patients for PBT. Applicants must meet four criteria for assessment including completion of CPP plans. This criterion relies upon the proton planning team at ABCPTR in South Australia or experienced overseas facilities to provide a CPP plan report. However, completing this process can be associated with clinically significant delays, resulting in the patient being unable to pursue PBT as a treatment option. Of note, the tripartite (RANZCR – Royal Australian and New Zealand College of Radiologists, ACPSEM – Australasian College of Physical Scientists and Engineers in Medicine and ASMIRT) PTSIG has published CPP planning guidelines,^13^ which recommends a timeframe of 7 days from patient data acquisition to a CPP plan report.

Developing in‐house CPP planning allows us to generate a CPP that better informs clinical decisions and filters out the time‐pressured and resource‐intensive MTOP application process if deemed not necessary. On occasion, cases have undergone a parallel process of in‐house comparisons and MTOP applications due to the urgency of treatments. Currently, the MTOP application requires the referring centre to provide a clinically acceptable photon plan. In our experience, the quality of this plan is imperative to enable a fair comparison to the proton plan. Particularly with a paediatric cohort, planning experience is vital.

Training and accreditation are essential when implementing new techniques and new technologies as part of standard clinical practice. Compared to external radiation therapy with megavoltage photon and electron beams, particle therapy technology is evolving rapidly; its planning techniques involve a set of distinct concepts. It is envisioned that as particle therapy becomes more widely available to the Australian community, that CPP may be carried out by a network of accredited centres, not just clinical proton therapy centres. This will be done to foster engagement in proton therapy services and to assist in sharing the workload of having to double plan proton therapy patients. This is now precedented by the Danish approach to decentralise the comparative planning workload^14^ for mutual benefits of referring site and treatment site.

The review and validation of eight cases (13% of total case) by experts in existing proton centres was very important, given hands‐on experience was limited within the team. It helped to ascertain the clinical deliverability of our treatment plans, and better inform us of proton‐specific considerations. It is intended that we receive further ongoing feedback from these centres.

### Future R&D opportunity

There are a number of aspects for future work to improve our comparative planning practice. Currently, our comparative planning is heavily focused on CNS in terms of treatment sites and a paediatric cohort in terms of age, aligning us with the current PBT indications as published in MSAC 1638 (the item number approved in November 2020).^15^ There is the potential to expand to other body sites, such as liver and lung.

With the increasing number of cases through the service, a centralised electronic database where all relevant information can be extracted for retrospective analysis is being considered.

While not having a PBT facility onsite can be seen as a limitation, there are R&D opportunities that our team is exploring. This includes automated planning and dual‐energy CT to better resolve stopping power ratio for different tissue types and its impact on different dose calculation algorithms.

## Conclusion with recommendations

Since 2019, Peter Mac has gained significant experience in CPP planning. This will support any future proposals for PBT in Victoria and enable us to potentially be a site for CPP if patient selection for proton treatments at ABCPTR is decentralised.

Acknowledging that our service has limitations and needs further development, this report will hopefully aid other centres looking to develop a similar service. A key principle of our team is to work collaboratively to contribute towards the development of particle therapy in Australia.

Based on our experience and available literature,^14^, ^16^ it is desirable to implement a national training and accreditation scheme for CPP treatment planning. We recommend the following items as a *minimum* set of requirements for training and accreditation of non‐proton therapy centres to perform comparative planning for patient eligibility assessment:

- The nominated lead RO, RT and ROMP receiving on‐site training at an experienced proton therapy centre that hosts training and/or certification programmes;
- In‐house training for multidisciplinary working group including RO/ROMP/RT within local site and documentation of such training record;
- Contour and Plan review by experienced proton therapy planning centres (either overseas or domestic) for a set of plans across body sites and tumour streams, then obtain a written form of satisfactory plan quality by comparison with the expert sets;
- CPD including training courses and/or structured education workshops provided by the vendors, well‐established proton centres, or scientific organisations.

## Conflict of Interest

The authors declare no conflict of interest.

## Ethics statement

This study with reporting the audit results of photon‐proton comparison results is in compliance with the ethics policy of our institution.

## Data Availability

The data that support the findings of this study are available on request from the corresponding author. The data are not publicly available due to privacy or ethical restrictions.
